# A protective role for periostin and TGF-β in IgE-mediated allergy and airway hyperresponsiveness

**DOI:** 10.1111/j.1365-2222.2011.03840.x

**Published:** 2011-08-22

**Authors:** E D Gordon, S S Sidhu, Z-E Wang, P G Woodruff, S Yuan, M C Solon, S J Conway, X Huang, R M Locksley, J V Fahy

**Affiliations:** 1 Division of Pulmonary and Critical Care MedicineSan Francisco, CA, USA; 2 Cardiovascular Research InstituteSan Francisco, CA, USA; 3 Microbiology and ImmunologySan Francisco, CA, USA; 4 Howard Hughes Medical Institute, University of CaliforniaSan Francisco, CA, USA; 5 Cardiovascular Development Group, Herman B Wells Center for Pediatric Research, Indiana University School of MedicineIndianapolis, IN, USA; 6 Lung Biology Center, University of CaliforniaSan Francisco, CA, USA

**Keywords:** airway hyperresponsiveness, asthma, IgE, mouse model, mucus metaplasia, peribronchial fibrosis, periostin, TGF-β

## Abstract

**Background:**

The pathophysiology of asthma involves allergic inflammation and remodelling in the airway and airway hyperresponsiveness (AHR) to cholinergic stimuli, but many details of the specific underlying cellular and molecular mechanisms remain unknown. Periostin is a matricellular protein with roles in tissue repair following injury in both the skin and heart. It has recently been shown to be up-regulated in the airway epithelium of asthmatics and to increase active TGF-β. Though one might expect periostin to play a deleterious role in asthma pathogenesis, to date its biological role in the airway is unknown.

**Objective:**

To determine the effect of periostin deficiency on airway responses to inhaled allergen.

**Methods:**

*In vivo* measures of airway responsiveness, inflammation, and remodelling were made in periostin deficient mice and wild-type controls following repeated intranasal challenge with *Aspergillus fumigatus* antigen. *In vitro* studies of the effects of epithelial cell-derived periostin on murine T cells were also performed.

**Results:**

Surprisingly, compared with wild-type controls, periostin deficient mice developed increased AHR and serum IgE levels following allergen challenge without differences in two outcomes of airway remodelling (mucus metaplasia and peribronchial fibrosis). These changes were associated with decreased expression of TGF-β1 and Foxp3 in the lungs of periostin deficient mice. Airway epithelial cell-derived periostin-induced conversion of CD4^+^ CD25^−^ cells into CD25^+^, Foxp3^+^ T cells *in vitro* in a TGF-β dependent manner.

**Conclusions and Clinical Relevance:**

Allergen-induced increases in serum IgE and bronchial hyperresponsiveness are exaggerated in periostin deficient mice challenged with inhaled aeroallergen. The mechanism of periostin's effect as a brake on allergen-induced responses may involve augmentation of TGF-β-induced T regulatory cell differentiation.

## Introduction

Asthma is a common disease characterized by reversible airflow obstruction, airway hyperresponsiveness (AHR), airway inflammation, mucus hypersecretion and sub-epithelial fibrosis 1. The cellular and molecular mechanisms underlying asthma remain incompletely understood, but new insights are being uncovered from the application of genomic technologies to human airway biospecimens. For example, in high-density microarray studies of gene expression in the airway epithelium in asthmatic subjects and healthy controls, we recently found that periostin is among the most highly up-regulated genes in asthma and that its expression in airway epithelial cells is regulated by interleukin 13 (IL-13), a Th2 cytokine 2. We went on to show that periostin gene expression in airway epithelial cells is a marker of an asthma subphenotype driven by excessive Th2-type inflammation and characterized by high levels of serum IgE, systemic and lung eosinophilia, increased thickness of the reticular basement membrane and responsiveness to corticosteroids 3.

Periostin is a matricellular protein first identified in osteoblasts and later found more widely expressed in mesenchymal cells in other organs 4–7. Periostin null mice show aberrant type 1 collagen fibrillogenesis in skin and poor integrity of the periodontal ligament in response to mechanical stress 8, 9. Further studies demonstrate the essential role of periostin in tissue repair in both the skin and heart following injury 10–12. Its interaction with type 1 collagen, fibronectin and tenascin C as well as integrins, αvβ3 and αvβ5, within the extracellular matrix (ECM) are thought to underlie its function in ECM organization and tissue repair 13–15. We have demonstrated that in airway epithelial cell culture models periostin increases levels of active TGF-β and that it has roles in regulating collagen synthesis and collagen gel elasticity 16. To begin to explore the biological roles of periostin in the airway in asthma, we took advantage of a periostin deficient mouse previously described 8 to determine how deficiency of periostin modulates airway responses to allergic airway inflammation. We anticipated that allergen challenge in mice would up-regulate periostin in the airway and model our previous findings in human asthma. Because we have found that periostin up-regulates TGF-β, we further anticipated that upregulation of lung periostin in these mice would be associated with an increase in TGF-β. TGF-β has multiple effects in the lung, including anti-inflammatory activity attributable to its induction of T regulatory cells and pro-fibrotic activity attributable to its effects on fibroblasts 17–23, so that its influence on asthma outcomes, such as AHR, airway eosinophilia and airway remodelling (peribronchial fibrosis and epithelial mucins), can vary depending on the specifics of the mouse model and the mechanism of TGF-β manipulation. Thus, we included a range of outcomes in our study design, including markers of AHR, eosinophils, T regulatory cells, peribronchial fibrosis and epithelial mucins.

Our results provide new insights into the roles of periostin in allergic airway inflammation.

## Materials and methods

### Mice

By replacing the translation start site and first exon with a lacZ reporter gene, 129SJv;C57BL/6 periostin deficient mice (*Pn*^*−/−*^) were generated 8. Similar numbers of male and female 12–13 week-old *Pn*^*−/−*^ mice and littermate wild-type controls were used in this study. Initial studies were performed in mixed background mice backcrossed into a C57BL/6 background for three generations (F3). However, to guard against the possibility that backcrossing for three generations may yield spurious results in some outcomes of allergen-induced inflammation 24, 25, we also performed experiments in mice backcrossed into the C57BL/6 background for six generations; these F6 mice were also in an IL-4 reporter background (4get) to enable study of IL-4 expressing cells 26. Mice were bred and maintained under specific pathogen-free conditions in the Laboratory Animal Resource Center at the University of California, San Francisco. The Committee on Animal Research at the University of California, San Francisco approved the use of mice for these experiments.

### Aspergillus antigen sensitization protocol

A mouse model of allergic lung disease was established using methods described previously with minor modifications 27. In brief, isoflurane anesthetized mice were given 100 μg (40 μl of saline) of *Aspergillus fumigatus* (Hollister-Stier Laboratories, Spokane, WA, USA), or 40 μl of normal saline alone applied to the nostrils using a micropipette with the mouse held in the supine position. After three treatments per week for 3 weeks, mice were killed 48 h after the last intranasal challenge.

### Airway hyperresponsiveness measurements

Forty-eight hours after the last challenge, mice were anaesthetized with ketamine (100 mg/kg of body weight) and xylazine (10 mg/kg). A tracheostomy was performed, and a tubing adaptor (20 gauge) was used to cannulate the trachea. The mice were then attached to a rodent ventilator and pulmonary mechanics analyzer (FlexiVent; SCIREQ Inc., Montreal, Canada) and ventilated at a tidal volume of 9 mL/kg, a frequency of 150 breaths/min, and 2 cm H_2_O positive end-expiratory pressure. Mice were paralysed with pancuronium (0.1 mg/kg intraperitoneally). A 27-gauge needle was placed in the tail vein, and measurements of airway mechanics were made continuously using the forced oscillation technique. Mice were given increasing doses of acetylcholine (0.1, 0.3, 1, 3 and 9.6 μg/g of body weight) administered through the tail vein to generate a concentration-response curve as previously described 28.

### Broncho-alveolar lavage cell counts

Lungs were subjected to lavage five times with 0.8 mL of phosphate-buffered saline (PBS). After centrifugation (200 g, 5 min), the cell pellet was resuspended in normal saline after lysis of red blood cells. Total cells were counted with a hemacytometer. Cytospin preparations were stained with a HEMA 3 stain set (Fisher Scientific, Pittsburgh, PA, USA), and broncho-alveolar lavage (BAL) fluid cell differential percentages were determined based on light microscopic evaluation of >300 cells/slide 29.

### Lung histology and immunohistochemistry

After lavage, lungs were inflated with 10% buffered formalin to 25 cm H_2_O of pressure. Multiple paraffin-embedded 5-μm sections of the entire mouse lung were prepared and stained with hematoxalin and eosin for regular morphology, with periodic acid-Schiff (PAS) for evaluation of mucus production, or with Sirius red for evaluation of fibrosis. Immunostaining was performed on histological lung sections using antibodies to detect periostin (rabbit polyclonal antibody at 1 : 3000 dilution; gift from Simon Conway, Indiana University School of Medicine, IN, USA). Histological sections were deparaffinized, rehydrated, incubated in 3% hydrogen peroxide/absolute methanol for 10 min and then blocked with 5% goat serum for 30 min at 23°C. Sections were then blotted and incubated in primary antibody diluted in 5% goat serum/0.3% Tween 20/PBS for 1 h at 23°C. Sections were then incubated for 1 h at 23°C in biotinylated donkey anti-rabbit (1 : 10 000, Jackson ImmunoResearch, Westgrove, PA, USA). Next, sections were incubated in ABC reagent (Vector Laboratories, Burlingame, CA, USA) for 1 h at 23°C, followed by DAB Plus reagent (Zymed, San Francisco, CA, USA) for 10 min and counterstaining with Gill's no. 3 hematoxylin.

### Quantitative measurement of epithelial mucin stores and peribronchial fibrosis using stereology

Design-based stereology was applied to histological sections of mouse lungs using a point and intercept-counting technique using an integrated microscope (Olympus, Albertslund, Denmark), video camera (JVC Digital Color; JVC, Tatstrup, Denmark), automated microscope stage, and computer (Dell Optiplex GS270 PC running Computer-Assisted Stereology Toolbox software; Olympus, Albertslund, Denmark) 2. A line segment grid was superimposed on systematically randomly selected microscope fields. Points overlying staining of interest were counted along with intersections of test lines with basal lamina. An investigator blinded to the experimental conditions recorded all measurements. The volume of interest was calculated by quantification of the volume of staining referenced to the surface area of basal lamina surveyed (cubic micrometre per square micrometre). Measures of epithelial mucin stores were made using the technique applied to approximately 250 random sections per mouse (40× magnification) of mouse lung stained with PAS. Peribronchial fibrosis measures were made using the technique applied to 100 random sections per mouse (60× magnification) of mouse lung stained with Sirius red. Measures of peribronchial fibrosis were made in airways of 100–300 μm diameter to avoid bias that might be introduced from sampling larger airways.

### Cell culture

Spleens from 6–8 week-old C57BL6 mice were harvested, manually dissociated and passed through a 70-μm strainer in DMEM containing 10% fetal calf serum (FCS) (Gibco, Carlsbad, CA, USA). Cells were incubated with allophycocyanin (APC)-Alexa Fluor 780–anti-CD4 (RM4-5) and phycoerytherin (PE)-anti-CD25 (BD Bioscience, San Diego, CA, USA) in 2% FCS-PBS for 30 min at 4°C, washed and resuspended in 2% FCS-PBS. CD4^+^ CD25^−^ cells were then purified using a MoFlo XDP High-Speed Cell Sorter (Beckman Coulter, Brea, CA, USA). CD4^+^ CD25^−^ cells were stimulated for 4 days with plate-bound anti-CD3 (2 μg/mL; 145-2C11, NA/LE, BD Bioscience), soluble anti-CD28 (2 μg/mL; BD Bioscience) and recombinant human IL-2 (20 U/mL, NCI Preclinical Repository, Frederick, MD, USA) and were cultured at 10^6^ cells/mL with recombinant human TGF-β1 (10 ng/mL, Humanzyme, Chicago, IL, USA) and recombinant human periostin (40 ng/mL, R&D Systems, Minneapolis, MN, USA). CD4^+^ CD25^−^ cells were also stimulated as above with plate-bound anti-CD3, soluble anti-CD28, and recombinant human IL-2 and co-cultured for 4 days with a human airway epithelial cell line (Beas2B) that contains a stably transfected human recombinant periostin expression vector (B2BPN) or a control vector (B2BCTL) 16 grown in air-liquid interface at 200 000 cells/mL in the presence or absence of the TGF-β1 receptor kinase inhibitor (SB 431542, 10 nM, Sigma-Aldrich, St. Louis, MO, USA) or the pan-TGF-β blocking antibody (50 μg/mL, AB-100-NA, R&D Systems). T cells were harvested for flow cytometic analysis and RT-PCR.

### Flow cytometric analyses

Flow cytometric analyses (FACS) were performed on whole lung single-cell suspensions 30. Lungs were perfused transcardially with 20 mL PBS, and the left lung was removed, mechanically dissociated and cells passed through a 70-μm filter to generate single-cell suspensions. Single-cell suspensions were washed in FACS buffer (PBS, 3% FCS, 1 mg/L NaN_3_) and incubated for 30 min on ice with the antibody to surface marker APC-Alexa Fluor 780–anti-CD4 (RM4-5; BD Biosciences). Cells were resuspended in 1 mg/mL 4′,6-diamidino-2-phenylindole to exclude dead cells. Samples were acquired on an LSRII flow cytometer (BD Biosciences) and analysed using FlowJo software (Tree Star, Ashland, OR, USA). Flow cytometric analysis was also performed on cultured mouse CD4^+^ CD25^−^ splenocytes. Cells were washed in 2% FCS-PBS and incubated for 30 min on ice with PE-anti-CD25 (BD Biosciences). Cells were washed in 2% FCS-PBS and samples were acquired on FACSCalibur (BD Biosciences) and analysed using FlowJo software (Tree Star).

### Western blot analysis

Whole lung tissue was homogenized in radioimmunoprecipitation assay buffer (150 mM NaCl, 1% Triton X-100, 0.1% SDS, 50 mM Tris-HCl pH 7.5) containing a cocktail of Complete Mini (proteinase inhibitors) and PhosSTOP (Roche, Mannheim, Germany). Protein concentrations were measured with BCA Protein Assay (Thermo Scientific, Rockford, IL, USA). A 20 μg of protein samples were electrophoresed under reducing conditions in 10% Tris-HCl Ready Gels (Biorad, Hercules, CA, USA) and transferred to Hybond nitrocellulose paper (Amersham, Piscataway, NJ, USA). Non-specific binding sites were blocked by washing blots in 5% non-fat milk (Nestle) in PBS containing 0.1% Tween20 (PBST; Sigma) for 1 h with shaking. Blots were incubated with primary antibody overnight at 4°C [rabbit anti-periostin 1 : 3000 dilution (a gift from Simon Conway, Indiana University, Indianapolis, IN, USA); mouse anti-GAPDH 1 : 4000 dilution (Ambion, Austin, TX, USA)], washed in PBST and incubated with HRP conjugated secondary antibody for 1 h at room temperature. Blots were washed again before visualization of immunocomplexes using electrochemiluminescence plus Western blotting kit and exposing to Hyperfilm (GE Healthcare, Piscataway, NJ, USA).

### Enzyme-linked immunosorbent assays

Enzyme-linked immunosorbent assays (ELISA) were performed on serum and whole lung homogenates. Sera were obtained from blood collected by cardiac puncture from mice after airway responsiveness measurements. Total serum IgE levels were measured using microplates coated with anti-mouse IgE (R35-72; BD Biosciences). Diluted serum samples were added to each well, and the bound IgE was detected with biotinylated anti-mouse IgE (R35-118; BD Biosciences). Colour development was achieved using streptavidin-conjugated horseradish peroxidase (BD Biosciences) followed by addition of horseradish peroxidase substrate (TMB; BD Biosciences). Whole lung homogenates were prepared as described above for Western blot analysis, and 20 μg of total protein (as determined by BCA Protein Assay described above) was diluted in 100 μl of reagent diluent according to manufacturer's protocol and assayed in duplicate for total mouse TGF-β1 using the R&D Systems mouse TGF-β1 Duoset ELISA Kit (Minneapolis, MN, USA). Optical densities were obtained at 450 nm on a SpectraMax Plate Reader (Molecular Devices, Sunnyvale, CA, USA) and results were analysed using SoftMax Pro V5.4 Software (Molecular Devices).

### RNA extraction and RT-PCR

The RNA was extracted from whole lung and thoracic lymph node homogenates using RNeasy mini-kits (Qiagen, Valencia, CA, USA) 31, and two-step qPCR was performed 2. Briefly, cDNA synthesis was carried out using 20 ng of total RNA and Superscript III (Invitrogen, Carlsbad, CA, USA) with random hexamers for priming. Multiplex pre-amplification was performed using one-fifth of the resultant cDNA, Advantage 2 Polymerase (Clontech, Mountain View, CA), and 5 pmol of each outflanking primer. Real-time PCR gene quantification was then performed on the amplified cDNA by using TaqMan probes (Applied Biosystems, Foster City, CA) and Universal Master Mix (Platinum Quantitative PCR SuperMix-UDG with ROX; Invitrogen). Transcript quantification was run on an ABI Prism 7900 Sequence Detection System (Applied Biosystems). Normalization was performed using Genorm Software and housekeeping genes GAPDH, RPS9 and PPIA.

### Statistical analyses

All numerical data were calculated as mean ± SEM and analysed using GraphPad Prism Software (LaJolla, CA, USA). Data were not transformed prior to analysis, except for PCR data, which were log transformed. Differences between experimental conditions were first assessed by one-way anova for each measure. Wherever group differences were confirmed by the anova procedure with a significance level of 0.05, we conducted post hoc Student *t-*tests for between group comparisons. To analyse the acetylcholine dose response curves, we used two-way anova (because there are two independent variables: treatment group and dose of acetylcholine) followed by Bonferonni post hoc tests when group differences were significant (*P* < 0.05).

## Results

### Aspergillus antigen airway challenge induces periostin protein expression in the lung

**Fig. 1:**
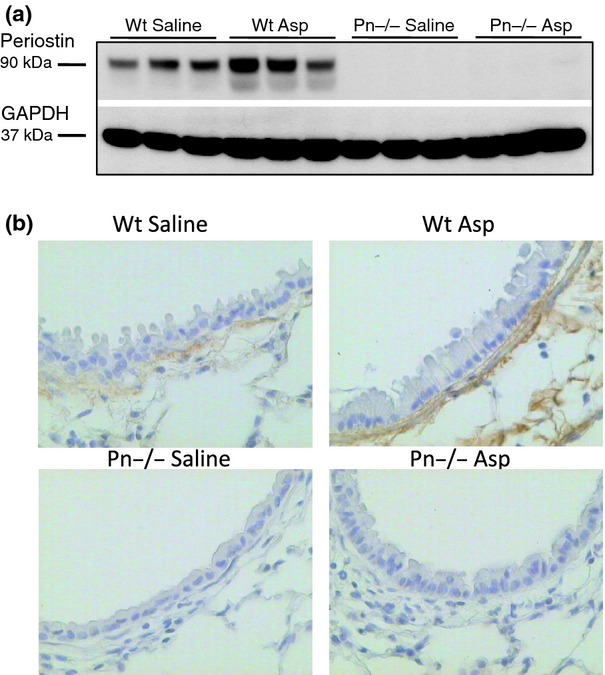
Periostin protein increases in the lung following airway challenge with *Aspergillus* antigen. (a) Periostin protein expression in the lungs of wild-type (Wt) and periostin deficient (*Pn*^*−/−*^) mice was determined by immunoblotting for periostin protein in lung extracts from saline or *Aspergillus* (Asp) antigen-challenged mice (F3). (b) Immunohistochemistry for periostin protein expression was performed on lung sections from Wt or *Pn*^*−/−*^ mice treated with saline or Asp antigen (F3). Magnification: 40×.

To determine if *Aspergillus* antigen challenge up-regulates periostin protein expression in the lung, we evaluated periostin protein expression by immunoblot of whole lung homogenates and by immunohistochemistry on whole lung sections from periostin deficient mice and wild-type 129;C57BL/6 controls (F3). As expected, no periostin protein was detectable in periostin deficient mice before or after *Aspergillus* antigen challenge (Figs 1a and b). However, periostin expression increased following challenge with *Aspergillus* antigen in wild-type mice (Figs 1a and b). Notably, periostin was not found in epithelial cells themselves but in the sub-epithelial space, consistent with the immunolocalization pattern we found previously in human airway mucosal biopsies and with our previous findings that periostin is expressed in airway epithelial cells but secreted rapidly in a basal direction into the matrix 16.

### Periostin deficient mice have increased airway hyperreactivity and higher systemic IgE responses following airway challenge with Aspergillus antigen

In F3 mice we found similar levels of airway responsiveness to acetylcholine in periostin null mice and littermate controls under non-challenged conditions, but the periostin null mice were significantly more reactive to acetylcholine following challenge with *Aspergillus* antigen (Supporting Information Fig. S1a). In addition, periostin null mice and controls had similar serum IgE levels under non-challenged conditions, but the periostin null mice had significantly higher serum IgE levels following airway challenge with *Aspergillus* antigen (Supporting Information Fig. S1b). Furthermore, the periostin null mice demonstrated no differences in BAL cell counts compared with wild-type littermate controls following allergen challenge (Supporting Information Fig. S1c).

**Fig. 2:**
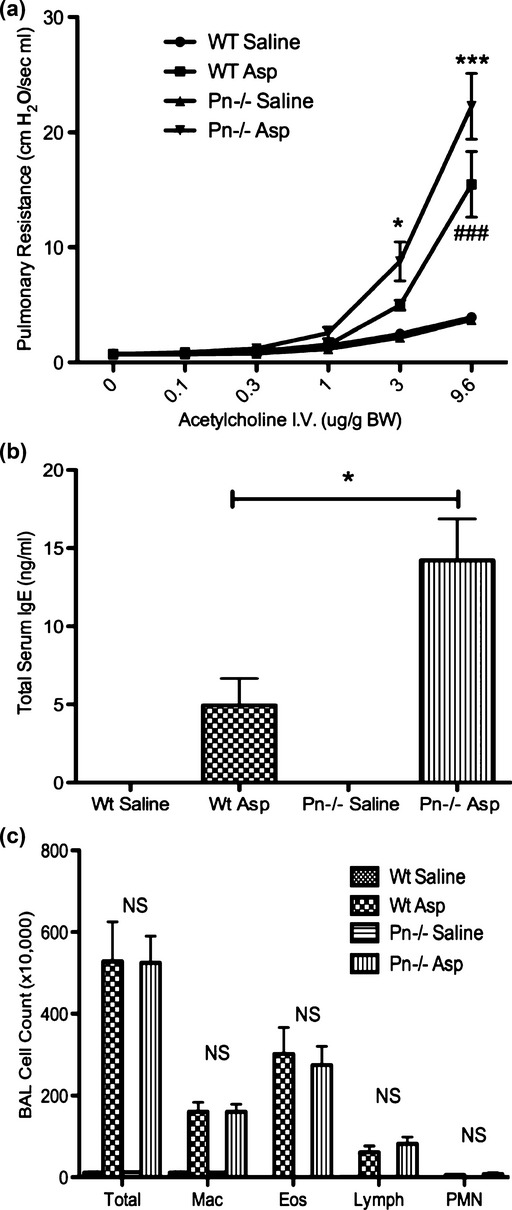
Periostin deficient mice have increased airway hyperreactivity and higher systemic IgE responses following airway challenge with *Aspergillus* antigen. (a) Airway reactivity to intravenously administered acetylcholine is similar in unchallenged periostin deficient (*Pn*^*−/−*^) mice (F6) and wild-type (Wt) mice, but acetylcholine reactivity following *Aspergillus* (Asp) challenge is more severe in *Pn*^*−/−*^ mice (F6). *P < 0.05, ***P < 0.001 for Wt Asp vs. *Pn*^*−/−*^ Asp. ###*P* < 0.001 Wt saline vs. Wt Asp. (b) Serum IgE levels are similar in *Pn*^*−/−*^ mice (F6) and wild-type mice, but IgE levels following Asp challenge are more severe in *Pn*^*−/−*^ mice (F6). *P < 0.05. (c) Total cell numbers in broncho-alveolar lavage (BAL) are similar in *Pn*^*−/−*^ mice (F6) and wild-type mice, and increased markedly following Asp challenge with increases in macrophages, eosinophils and lymphocytes; these increases occurred similarly in *Pn*^*−/−*^ (F6) and wild-type mice. Results represent mean ± SEM for 5–7 mice per group (F6).

Because of the possibility that insufficient backcrossing could influence physiological and inflammatory outcomes in mouse models of asthma 24, 25, we repeated the *Aspergillus* allergen protocol in mice backcrossed six times into a C57BL/6 background (F6). In these repeat experiments we simultaneously backcrossed the mice into a 4get background, because the IL-4-IRES-eGFP (4get) reporter allows monitoring of IL-4 expressing cell trafficking and cell sorting. As with the F3 mice, the F6 mice showed similar levels of airway responsiveness to acetylcholine in periostin null mice and littermate controls under non-challenged conditions. However, the periostin null mice were significantly more reactive to acetylcholine following challenge with *Aspergillus* antigen (Fig. 2a). In addition, periostin null mice and controls had similar serum IgE levels under non-challenged conditions, but the periostin null mice had significantly higher serum IgE levels following airway challenge with *Aspergillus* antigen (Fig. 2b). Finally, we did not find a significant difference in the allergen-induced increase in BAL cell counts in the periostin null mice compared with controls (Fig. 2c).

### GFP^+^, IL-4 producing cells and GFP^+^ CD4^+^ cells increase similarly in periostin deficient mice and wild-type controls following airway challenge with *Aspergillus* antigen

**Fig. 3:**
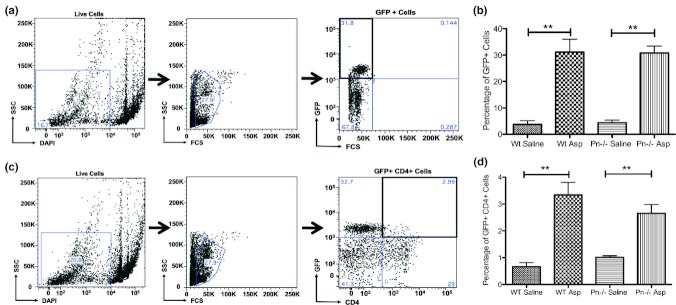
Periostin deficient mice and wild-type controls have a similar increase in *GFP*^+^ *IL-4* producing cells and *GFP*^*+*^ *CD4*^*+*^ cells within the lung following airway challenge with *Aspergillus* antigen. Schematic scheme showing a representative flow cytometric analysis for GFP^+^ cells (a) or GFP^+^ CD4^+^ cells (c) performed on whole lung single-cell suspensions from periostin deficient mice (*Pn*^*−/−*^) in C57BL/6 4get background (F6) and wild-type (Wt) littermate controls. There is a similar increase in GFP-producing cells (b) and GFP^+^ CD4^+^ cells (d) in the lungs of *Pn*^*−/−*^ mice and Wt littermate controls following *Aspergillus* (Asp) antigen challenge. Results represent mean ± SEM for 3–5 mice (F6) per group. **P < 0.01.

To determine if changes in AHR and serum IgE in the periostin deficient mice following allergen challenge might be explained by differences in the numbers of IL-4-producing cells within the lung tissue, we performed flow cytometric analysis on whole lung single-cell suspensions made from periostin null and wild-type littermate controls in the C57BL/6 4get background (F6) following *Aspergillus* antigen challenge (Fig. 3a). In this model system, IL-4-producing cells express the GFP protein. We found that following *Aspergillus* antigen challenge, GFP-producing cells accumulated similarly in the lungs of both periostin null mice and wild-type littermate controls (Fig. 3b). In addition, the subset of GFP^+^ cells that were positive for CD4 surface staining also increased similarly in the lungs of periostin deficient mice and wild-type littermate control (Fig. 3d). There were no differences in numbers of lung dendritic cells detected in periostin deficient and wild-type mice following allergen challenge (data not shown). Furthermore, analysis of mRNA levels of IL-4, 5 and 13, within the draining lymph nodes of periostin null and wild-type control mice (F6) following allergen challenge demonstrated similar increases compared to saline-challenged mice (Supporting Information Figs S2a–c).

### Periostin deficient mice have decreased TGF-β1 and Foxp3 in the lung compared to wild-type littermate controls

**Fig. 4:**
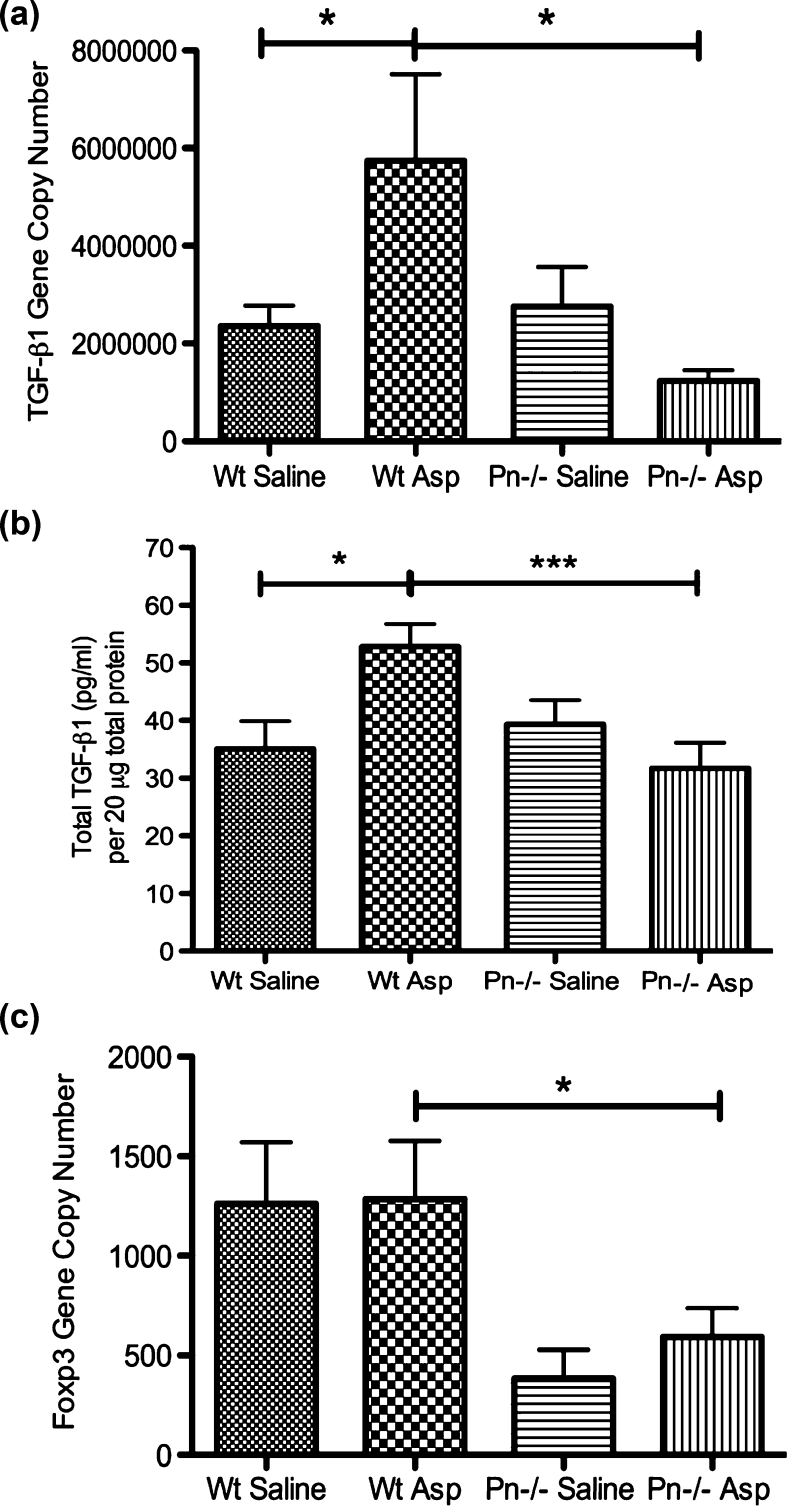
Periostin deficient mice have less TGF-β1 and Foxp3 gene transcript and TGF-β1 protein in the lung following *Aspergillus* antigen challenge. TGF-β1 and Foxp3 mRNA (F6) and TGF-β1 protein (F3) in lungs from saline and *Aspergillus* (Asp) antigen-challenged, wild-type (Wt) and *Pn*^*−/−*^ mice. (a) TGF-β1 transcripts increase in Wt mice following allergen challenge but do not in the *Pn*^*−/−*^ mice. (b) TGF-β1 protein per 20 μg of total protein increases in Wt mice following allergen challenge but does not in the *Pn*^*−/−*^ mice. (c) Foxp3 transcripts are decreased in *Pn*^*−/−*^ mice compared with wild-type (Wt) controls following allergen challenge. Results represent mean ± SEM for 3–5 mice per group. *P < 0.05, **P < 0.01, ***P < 0.001.

Periostin is known to increase TGF-β in airway epithelial cell culture 32, so we measured TGF-β1 gene expression and total protein in the lungs of periostin deficient mice and wild-type controls following allergen challenge. Consistent with our prior data, we found that periostin deficient mice have decreased TGF-β1 gene transcript and total protein in the lung following allergen challenge compared with wild-type controls (Figs 4a and b). TGF-β is known to induce the differentiation of T regulatory cells, so we measured Foxp3 (a transcription factor found in T regulatory cells) in whole lung. We found that Foxp3 gene expression was decreased in periostin null mice compared to littermate controls following *Aspergillus* antigen challenge (Fig. 4c). These findings suggest that periostin is a regulator of TGF-β1 *in vivo* during allergic lung inflammation. A decrease in TGF-β1 in lung likely explains the reduction in Foxp3 expression, a marker of T regulatory cells.

### Epithelial cell-derived periostin induces Foxp3^+^ T regulatory cell differentiation in a TGF-β dependent manner

**Fig. 5:**
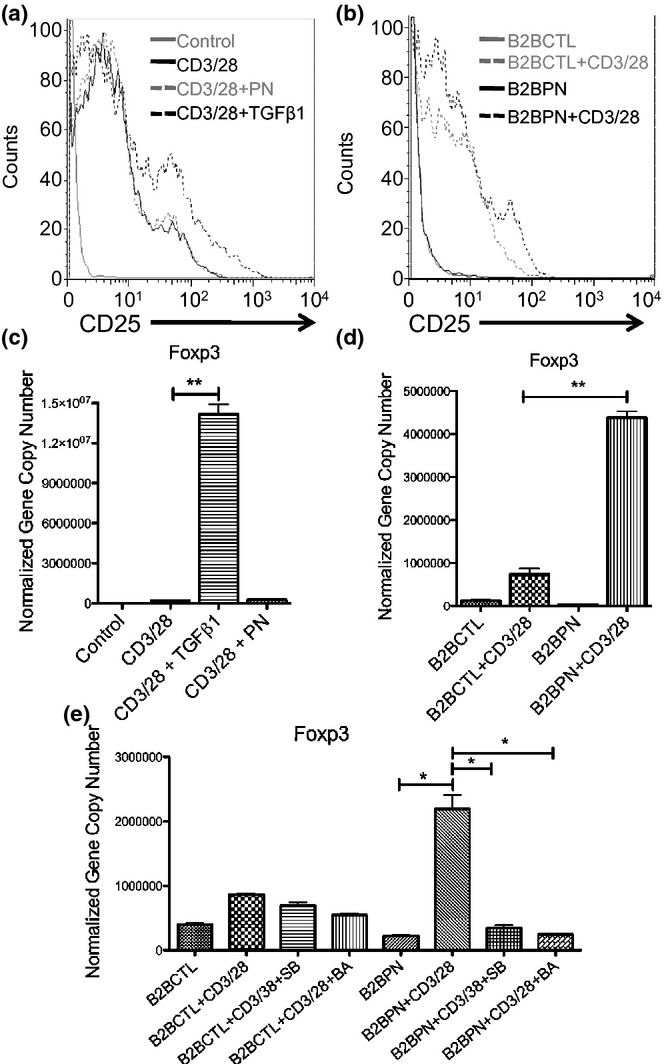
Epithelial cell-derived periostin induces Foxp3 expression in T cells in a TGF-β1 dependent manner. Recombinant human TGF-β1 (10 ng/mL) but not recombinant human periostin induces CD25 expression (a) and Foxp3 gene expression (c) in activated CD4^+^ CD25^−^ mouse T cells. Co-culture of activated CD4^+^ CD25^−^ mouse T cells with Beas2B cells over-expressing periostin (B2BPN) but not a control vector (B2BCTL) induces CD25 expression (b) and Foxp3 gene expression (d). The induction of Foxp3 gene expression in activated CD4^+^CD25^−^ mouse T cells co-cultured with B2BPN is blocked by treatment with the TGF-β1 receptor kinase inhibitor SB-431542 (SB) and the pan-TGF-β blocking antibody (BA) (e). Results represent mean ± SEM, *N* = 3. *P < 0.05, **P < 0.01.

We have previously demonstrated that epithelial cell-derived periostin increases active TGF-β in cultures of airway epithelial cells 16. TGF-β is known to exert anti-inflammatory effects in allergic airway disease via the conversion of CD4^+^ CD25^−^ to CD25^+^ T regulatory cells 33. We investigated if periostin can induce differentiation of T regulatory cells in a TGF-β dependent manner. Mouse CD4^+^ CD25^−^ T cells were isolated by flow cytometry, stimulated with anti-CD3, anti-CD28, and recombinant IL-2 and cultured with recombinant human TGF-β1, recombinant human periostin, or co-cultured with airway epithelial cells (Beas2B) which are stably transfected with a human periostin expression vector or a control vector 16. Following activation and stimulation with anti-CD3, anti-CD28, and recombinant IL-2, recombinant TGF-β1, but not recombinant periostin alone, induces an increase in surface expression of CD25 and gene expression of Foxp3 in CD4^+^ CD25^−^ T cells (Figs 5a and c). However, when co-cultured with airway epithelial cells over-expressing periostin, the CD4^+^ CD25^−^ T cells increased CD25 surface expression and Foxp3 gene expression (Figs 5b and d). The induction of Foxp3 expression in T cells co-cultured with airway epithelial cells over-expressing periostin is blocked by TGF-β signalling inhibition with the type I receptor kinase inhibitor SB 431542 and the pan-TGF-β antibody (Fig. 5e).

### Measures of epithelial mucin stores and peribronchial fibrosis increase similarly in periostin deficient mice and wild-type controls following airway challenge with *Aspergillus* antigen

**Fig. 6:**
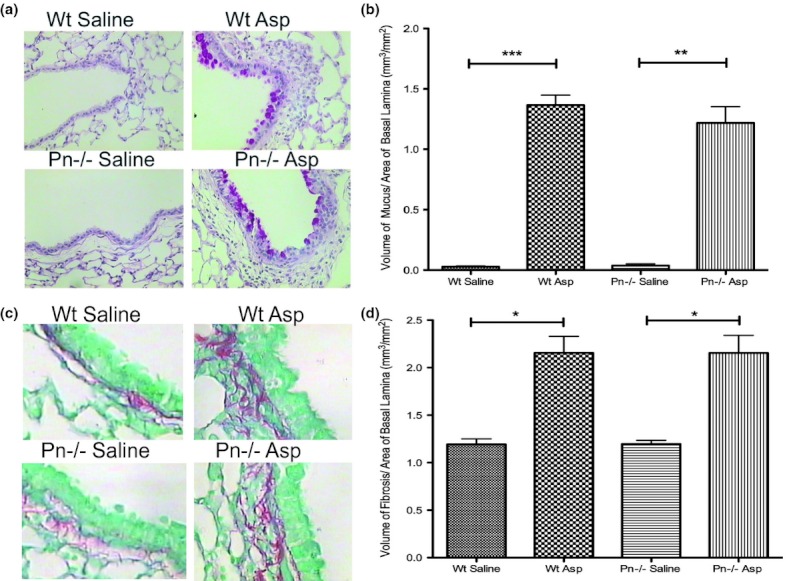
Periostin deficient mice develop increased epithelial mucin stores and peribronchial collagen deposition in response to *Aspergillus* antigen. (a) Representative periodic acid-Schiff (PAS)-stained sections from saline treated wild-type (Wt) or periostin deficient (*Pn*^*−/−*^) mice demonstrate minimal PAS-positive staining. *Aspergillus* (Asp) antigen treated Wt and *Pn*^*−/−*^ mice show magenta staining within epithelial cells, representing mucin stores. Magnification: 20×. (b) Epithelial mucin stores, represented as the volume of PAS-stained regions referenced to the area of epithelial basal lamina, increase markedly after Asp challenge in both *Pn*^*−/−*^ mice and wild-type mice (F3). Results represent mean ± SEM for 5–6 mice (F3) per group. (c) Representative Sirius red-stained sections from saline treated Wt or *Pn*^*−/−*^ mice demonstrate minimal collagen staining. Asp treated Wt and periostin *Pn*^*−/−*^ mice show increased peribronchial Sirius red staining representing airway fibrosis. Magnification: 20×. (d) Peribronchial fibrosis of small-medium sized airways (100–300 μm diameter), represented as the volume of Sirius red staining per basal lamina, increase after Asp challenge in both *Pn*^*−/−*^ mice and Wt mice (F3). Results represent mean ± SEM for 3–4 mice (F3) per group. Results represent mean ± SEM for (F3) per group. *P < 0.05, **P < 0.01, ***P < 0.001.

To determine if periostin affects epithelial mucin stores or peribronchial fibrosis, we quantified PAS staining in the epithelium and Sirius red staining of the peribronchial region. PAS staining showed that epithelial mucin stores increased significantly following *Aspergillus* antigen challenge in periostin deficient mice, but there was no difference in periostin deficient mice and wild-type controls (Figs 6a and b). Sirius red staining showed that peribronchial fibrosis also increased significantly following *Aspergillus* antigen challenge in periostin deficient mice with no significant difference compared to wild-type controls (Figs 6c and d).

## Discussion

Periostin expression is increased in the airways of asthmatic subjects 2, 34, but its role in asthma pathogenesis is unknown. Herein we explore the biological role of periostin in asthma using a mouse model and periostin deficient mice. We anticipated that periostin deficiency would protect from allergen-induced inflammation and hyperresponsiveness, but we found the opposite effect. Specifically, we found that compared to wild-type controls, periostin deficient mice have increased AHR and markedly increased serum IgE levels following repeated intranasal challenge with *A*. *fumigatus* antigen without differences in IL-4 producing-Th2 cells or in two outcomes of airway remodelling (epithelial mucin stores and peribronchial fibrosis). We also found evidence that periostin deficient mice have blunted TGF-β responses to allergen leading us to propose that the protective effects of epithelial cell-derived periostin are mediated by TGF-β-induced differentiation of T regulatory cells. In support of this hypothesis, we find that airway epithelial cell-derived periostin, but not recombinant periostin, induces conversion of CD4^+^ CD25^−^ T cells into CD25^+^ Foxp3^+^ T cells *in vitro* in a TGF-β dependent manner. These data suggest an important role for airway epithelial cell-derived periostin and local TGF-β activation on the regulation of allergic immune responses in the airway.

Periostin deficient mice challenged with *Aspergillus* allergen were significantly more hyperresponsive to acetylcholine than wild-type controls and demonstrated marked increases in systemic IgE responses. It should be noted that C57B6 mice traditionally demonstrate less hyperresponsiveness in response to allergen than other mouse strains 35, and the large changes in AHR reported herein likely relate to the aspergillus allergen model used. Aspergillus antigen challenge 36, 37 typically yields higher AHR measurements in C57BL6 mice than the ovalbumin model 38–40. The mechanism of the effects of periostin on AHR and serum IgE levels may relate to the role of locally produced TGF-β and its anti-inflammatory effects within the airway. For example, we recently reported that periostin is a product of airway epithelial cells that activates TGF-β in a mechanism involving matrix metalloproteinases 16. Consistent with those findings, we demonstrate that TGF-β1 gene expression and total protein are decreased in periostin deficient mice following allergen challenge.

A negative regulatory role for periostin and TGF-β in allergic airway responses is plausible. TGF-β is a complex and pleiotropic cytokine with numerous cellular functions, including pro and anti-inflammatory effects that depend on the context of its activation 16, 33, 41. TGF-β induces the development of CD4^+^ CD25^+^, Foxp3^+^ T regulatory cells 33, 42, which suppress IgE 43–45 and CD4^+^ CD25^−^ T cell proliferation 46–48 via IL-10 production. CD4^+^ CD25^+^ T regulatory cells have also been shown to significantly reduce AHR in mouse models of asthma 49–51. A reduction in T regulatory cells in the periostin deficient mice might therefore explain the enhanced serum IgE responses and AHR following airway allergen challenge in these mice. We found evidence for this mechanism in our experiments. TGF-β1 was decreased in the lungs of the periostin deficient mice following allergen challenge, as were Foxp3 transcripts. The reduction in Foxp3 transcripts suggests reduced numbers of T regulatory cells, and we would expect concomitant reductions in IL-4 producing CD4^+^ T effector cells or decreased levels of Th2 cytokines. The absence of these findings leads us to speculate that reductions in other TGF-β responsive cell types, such as Th17 or Th9 cells, might explain these findings.

In complimentary *in vitro* experiments, we demonstrate that epithelial cell-derived periostin, but not recombinant periostin alone, can promote development of CD25^+^ Foxp^+^ T cells from CD4^+^ CD25^−^ T cells. This suggests that while periostin plays a role in the development of a T regulatory cell phenotype in this model, other epithelial cell-derived factors such as latent TGF-β, matrix metalloproteinase, or epithelial integrins, are required for the induction of Foxp3 gene expression. Furthermore, we show that epithelial cell-derived periostin up-regulates Foxp3 gene expression in a mechanism dependent on TGF-β1 activity. While these data do not preclude the possibility that other cell types such as fibroblasts are also a source of periostin in the asthmatic lung, they suggest a role for epithelial cell-derived periostin in TGF-β-mediated regulation of airway immune responses via epithelial-T cell cross-talk.

We did not find differences in BAL eosinophilia between the periostin null mice and wild-type controls following *Aspergillus* antigen challenge, a finding that differs from recent reports that periostin deficiency exacerbates oesophageal eosinophilia in a mouse model of allergic oesophagitis 52. However, our experiments were performed in mice backcrossed into a C57BL/6 background, whereas the allergic oesophagitis studies were done in the 129SVEV background 52, and it is possible that eosinophil responses in different model systems are strain specific.

The effects of periostin deficiency on allergen-induced AHR and serum IgE occurred without any effect on allergen-induced changes in peribronchial fibrosis. Periostin has been implicated in mechanisms of wound repair and collagen fibrillogenesis 10, 12, 14; thus, periostin deficiency with a concomitant decrease in TGF-β1 might have been expected to inhibit allergen-induced airway fibrosis. For example, periostin null mice exhibit a significant decrease in the thickness of the collagenous dermal layer of the skin and display abnormal collagen fibrillogenesis and a reduced level of collagen cross-linking 10, 14. It is possible that non-TGF-β1 dependent pathways are recruited in the development of peribronchial fibrosis in this model of allergic inflammation. It is also possible that aberrations in peribronchial collagen matrix deposition or collagen cross-linking are indeed present in the periostin deficient mice following allergen challenge and may even contribute to the observed increase in bronchial hyperresponsiveness but that these differences are not readily measured using stereological techniques.

We found that airway challenge with *Aspergillus* allergen caused a large increase in epithelial mucin stores that was not affected by periostin deficiency. This result differs from a recent article showing that periostin deficiency results in enhanced ovalbumin-induced mucus production and increased mucin gene expression in the airway 53. Multiple differences in methods could explain these divergent outcomes for mucin outcomes in periostin deficient mice, including differences in allergen (*Aspergillus* antigen vs. ovalubumin), mouse strain (mixed vs. C56BL/6) and methods of quantification (semi-quantitative vs. stereology-based). In the systems we used, we found no evidence that periostin regulates airway epithelial mucin stores.

In summary, we find that periostin's role in the airway is to act as a brake on allergen-induced IgE production and AHR. The mechanism of this effect may be explained by periostin's regulation of TGF-β and the anti-inflammatory effects of TGF-β-induced T regulatory cell differentiation. Overall, our data provide support for a novel paradigm in which periostin mediates a role for the epithelium in regulating T cell responses to inhaled allergens.

## Supporting Information

**Fig. S1.** 129SJv;C57BL/6 (F3) Periostin deficient mice have increased airway hyperreactivity, systemic IgE responses, and BAL eosinophilia following airway challenge with *Aspergillus* antigen. (a) Airway reactivity to intravenously administered acetylcholine is similar in periostin deficient (*Pn*^*-/-*^) mice (F3) and wild type (Wt) mice, but acetylcholine reactivity following *Aspergillus* (Asp) challenge is more severe in *Pn*^*-/-*^ mice (F3). (b) Serum IgE levels are similar in *Pn*^*-/-*^ mice (F3) and Wt mice, but IgE levels following Asp challenge are more severe in *Pn*^*-/-*^ mice (F3). (c) Total cell numbers in bronchoalveolar lavage (BAL) are similar in *Pn*^*-/-*^ mice (F6) and Wt mice, and increased markedly following Asp challenge with increases in macrophages, eosinophils, and lymphocytes. Results represent mean ± SEM for 7-9 mice per group. **P* < 0.05, ***P* < 0.01, ****P* < 0.001.

**Fig. S2.** Periostin deficient mice and wild-type controls express similar levels of TH2 cytokines in draining thoracic lymph nodes following *Aspergillus* antigen challenge. mRNA for TH2 cytokines (IL-4, IL-5, and IL-13) in draining thoracic lymph nodes from saline and *Aspergillus* (Asp) antigen challenged, wild-type (Wt) and periostin deficient (*Pn*^−/−^) mice (F6). (a) IL-4 transcripts increase similarly in Wt mice and *Pn*^−/−^ mice (F6) following allergen challenge. (b) IL-5 transcripts increase similarly in Wt mice and *Pn*^−/−^ mice (F6) following allergen challenge. (c) IL-13 transcripts increase similarly in Wt mice and *Pn*^−/−^ mice (F6) following allergen challenge. Results represent mean ± SEM for 3–5 mice (F6) per group. **P* < 0.05, ***P* < 0.01.

## References

[1] Holgate ST (2008). Pathogenesis of asthma. Clin Exp Allergy.

[2] Woodruff PG, Boushey HA, Dolganov GM (2007). Genome-wide profiling identifies epithelial cell genes associated with asthma and with treatment response to corticosteroids. Proc Natl Acad Sci USA.

[3] Woodruff PG, Modrek B, Choy DF (2009). T-helper type 2-driven inflammation defines major subphenotypes of asthma. Am J Respir Crit Care Med.

[4] Emans PJ, Spaapen F, Surtel DA (2007). A novel *in vivo* model to study endochondral bone formation; HIF-1alpha activation and BMP expression. Bone.

[5] Suzuki H, Amizuka N, Kii I (2004). Immunohistochemical localization of periostin in tooth and its surrounding tissues in mouse mandibles during development. Anat Rec A Discov Mol Cell Evol Biol.

[6] Snider P, Hinton RB, Moreno-Rodriguez RA (2008). Periostin is required for maturation and extracellular matrix stabilization of noncardiomyocyte lineages of the heart. Circ Res.

[7] Kruzynska-Frejtag A, Wang J, Maeda M (2004). Periostin is expressed within the developing teeth at the sites of epithelial-mesenchymal interaction. Dev Dyn.

[8] Rios H, Koushik SV, Wang H (2005). Periostin null mice exhibit dwarfism, incisor enamel defects, and an early-onset periodontal disease-like phenotype. Mol Cell Biol.

[9] Rios HF, Ma D, Xie Y (2008). Periostin is essential for the integrity and function of the periodontal ligament during occlusal loading in mice. J Periodontol.

[10] Jackson-Boeters L, Wen W, Hamilton DW (2009). Periostin localizes to cells in normal skin, but is associated with the extracellular matrix during wound repair. J Cell Commun Signal.

[11] Dobaczewski M, Gonzalez-Quesada C, Frangogiannis NG (2010). The extracellular matrix as a modulator of the inflammatory and reparative response following myocardial infarction. J Mol Cell Cardiol.

[12] Shimazaki M, Nakamura K, Kii I (2008). Periostin is essential for cardiac healing after acute myocardial infarction. J Exp Med.

[13] Kii I, Nishiyama T, Li M (2010). Incorporation of tenascin-C into the extracellular matrix by periostin underlies an extracellular meshwork architecture. J Biol Chem.

[14] Norris RA, Damon B, Mironov V (2007). Periostin regulates collagen fibrillogenesis and the biomechanical properties of connective tissues. J Cell Biochem.

[15] Li G, Jin R, Norris RA (2010). Periostin mediates vascular smooth muscle cell migration through the integrins alphavbeta3 and alphavbeta5 and focal adhesion kinase (FAK) pathway. Atherosclerosis.

[16] Sidhu SS, Yuan S, Innes AL (2010). Roles of epithelial cell-derived periostin in TGF-beta activation, collagen production, and collagen gel elasticity in asthma. Proc Natl Acad Sci USA.

[17] Luo X, Ding Q, Wang M (2010). *In vivo* disruption of TGF-beta signaling by Smad7 in airway epithelium alleviates allergic asthma but aggravates lung carcinogenesis in mouse. PLoS ONE.

[18] Scherf W, Burdach S, Hansen G (2005). Reduced expression of transforming growth factor beta 1 exacerbates pathology in an experimental asthma model. Eur J Immunol.

[19] Alcorn JF, Rinaldi LM, Jaffe EF (2007). Transforming growth factor-beta1 suppresses airway hyperresponsiveness in allergic airway disease. Am J Respir Crit Care Med.

[20] Anthoni M, Wang G, Leino MS, Lauerma AI, Alenius HT, Wolff HJ (2007). Smad3 -signalling and Th2 cytokines in normal mouse airways and in a mouse model of asthma. Int J Biol Sci.

[21] Fattouh R, Midence NG, Arias K (2008). Transforming growth factor-beta regulates house dust mite-induced allergic airway inflammation but not airway remodeling. Am J Respir Crit Care Med.

[22] Nemeth K, Keane-Myers A, Brown JM (2010). Bone marrow stromal cells use TGF-beta to suppress allergic responses in a mouse model of ragweed-induced asthma. Proc Natl Acad Sci USA.

[23] Bottoms SE, Howell JE, Reinhardt AK, Evans IC, McAnulty RJ (2010). Tgf-Beta isoform specific regulation of airway inflammation and remodelling in a murine model of asthma. PLoS ONE.

[24] Kodama M, Asano K, Oguma T (2010). Strain-specific phenotypes of airway inflammation and bronchial hyperresponsiveness induced by epicutaneous allergen sensitization in BALB/c and C57BL/6 mice. Int Arch Allergy Immunol.

[25] Zhu W, Gilmour MI (2009). Comparison of allergic lung disease in three mouse strains after systemic or mucosal sensitization with ovalbumin antigen. Immunogenetics.

[26] Reese TA, Liang HE, Tager AM (2007). Chitin induces accumulation in tissue of innate immune cells associated with allergy. Nature.

[27] Mishra A, Weaver TE, Beck DC, Rothenberg ME (2001). Interleukin-5-mediated allergic airway inflammation inhibits the human surfactant protein C promoter in transgenic mice. J Biol Chem.

[28] Chen C, Huang X, Sheppard D (2006). ADAM33 is not essential for growth and development and does not modulate allergic asthma in mice. Mol Cell Biol.

[29] Koth LL, Rodriguez MW, Bernstein XL (2004). Aspergillus antigen induces robust Th2 cytokine production, inflammation, airway hyperreactivity and fibrosis in the absence of MCP-1 or CCR2. Respir Res.

[30] Price AE, Liang HE, Sullivan BM (2010). Systemically dispersed innate IL-13-expressing cells in type 2 immunity. Proc Natl Acad Sci USA.

[31] Dolganov GM, Woodruff PG, Novikov AA (2001). A novel method of gene transcript profiling in airway biopsy homogenates reveals increased expression of a Na+-K+-Cl- cotransporter (NKCC1) in asthmatic subjects. Genome Res.

[32] Sidhu SS, Leong S, Woodruff P, Fahy J (2009). Periostin a novel mediator of epithelial mesenchymal communication in asthma.

[33] Chen W, Jin W, Hardegen N (2003). Conversion of peripheral CD4 + CD25- naive T cells to CD4 + CD25 + regulatory T cells by TGF-beta induction of transcription factor Foxp3. J Exp Med.

[34] Yuyama N, Davies DE, Akaiwa M (2002). Analysis of novel disease-related genes in bronchial asthma. Cytokine.

[35] De Sanctis GT, Daheshia M, Daser A (2001). Genetics of airway hyperresponsiveness. J Allergy Clin Immunol.

[36] Schuh JM, Power CA, Proudfoot AE, Kunkel SL, Lukacs NW, Hogaboam CM (2002). Airway hyperresponsiveness, but not airway remodeling, is attenuated during chronic pulmonary allergic responses to Aspergillus in CCR4-/- mice. FASEB J.

[37] Blease K, Mehrad B, Standiford TJ (2000). Enhanced pulmonary allergic responses to Aspergillus in CCR2-/- mice. J Immunol.

[38] Presser K, Schwinge D, Wegmann M (2008). Coexpression of TGF-beta1 and IL-10 enables regulatory T cells to completely suppress airway hyperreactivity. J Immunol.

[39] Caceres AI, Brackmann M, Elia MD (2009). A sensory neuronal ion channel essential for airway inflammation and hyperreactivity in asthma. Proc Natl Acad Sci USA.

[40] Lim DH, Cho JY, Song DJ, Lee SY, Miller M, Broide DH (2009). PI3K gamma-deficient mice have reduced levels of allergen-induced eosinophilic inflammation and airway remodeling. Am J Physiol Lung Cell Mol Physiol.

[41] Bettelli E, Carrier Y, Gao W (2006). Reciprocal developmental pathways for the generation of pathogenic effector TH17 and regulatory T cells. Nature.

[42] Sun CM, Hall JA, Blank RB (2007). Small intestine lamina propria dendritic cells promote de novo generation of Foxp3 T reg cells via retinoic acid. J Exp Med.

[43] Jeannin P, Lecoanet S, Delneste Y, Gauchat JF, Bonnefoy JY (1998). IgE versus IgG4 production can be differentially regulated by IL-10. J Immunol.

[44] Satoguina JS, Weyand E, Larbi J, Hoerauf A (2005). T regulatory-1 cells induce IgG4 production by B cells: role of IL-10. J Immunol.

[45] Akdis CA, Blesken T, Akdis M, Wuthrich B, Blaser K (1998). Role of interleukin 10 in specific immunotherapy. J Clin Invest.

[46] Taylor A, Akdis M, Joss A (2007). IL-10 inhibits CD28 and ICOS costimulations of T cells via src homology 2 domain-containing protein tyrosine phosphatase 1. J Allergy Clin Immunol.

[47] Taylor A, Verhagen J, Akkoc T (2009). Akdis CA, IL-10 suppresses CD2-mediated T cell activation via SHP-1. Mol Immunol.

[48] Akdis CA, Joss A, Akdis M, Faith A, Blaser K (2000). A molecular basis for T cell suppression by IL-10: CD28-associated IL-10 receptor inhibits CD28 tyrosine phosphorylation and phosphatidylinositol 3-kinase binding. FASEB J.

[49] Burchell JT, Wikstrom ME, Stumbles PA, Sly PD, Turner DJ (2009). Attenuation of allergen-induced airway hyperresponsiveness is mediated by airway regulatory T cells. Am J Physiol Lung Cell Mol Physiol.

[50] McGee HS, Agrawal DK (2009). Naturally occurring and inducible T-regulatory cells modulating immune response in allergic asthma. Am J Respir Crit Care Med.

[51] Joetham A, Takeda K, Taube C (2007). Naturally occurring lung CD4(+)CD25(+) T cell regulation of airway allergic responses depends on IL-10 induction of TGF-beta. J Immunol.

[52] Blanchard C, Mingler MK, McBride M (2008). Periostin facilitates eosinophil tissue infiltration in allergic lung and esophageal responses. Mucosal Immunol.

[53] Sehra S, Yao W, Nguyen ET (2011). Periostin regulates goblet cell metaplasia in a model of allergic airway inflammation. J Immunol.

