# Supplementary figures and images for: A protective role for periostin and TGF-β in IgE-mediated allergy and airway hyperresponsiveness
Source: Clin Exp Allergy. 2011 Aug 22;42(1):144–55. doi: 10.1111/j.1365-2222.2011.03840.x (PMC3271792; doi:10.1111/j.1365-2222.2011.03840.x)

## Slide 1
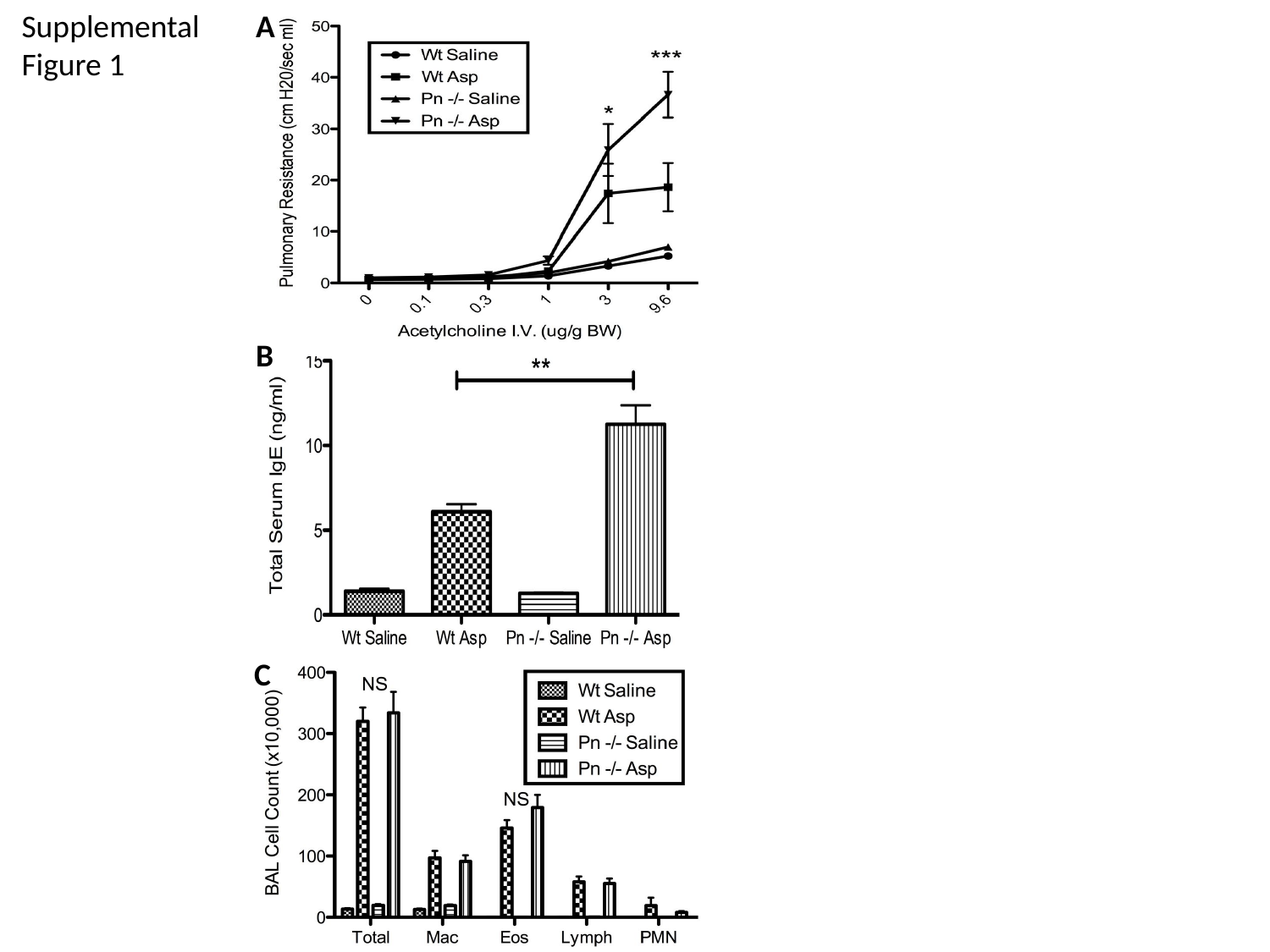

Supplemental
Figure 1
A
B
C

## Slide 2
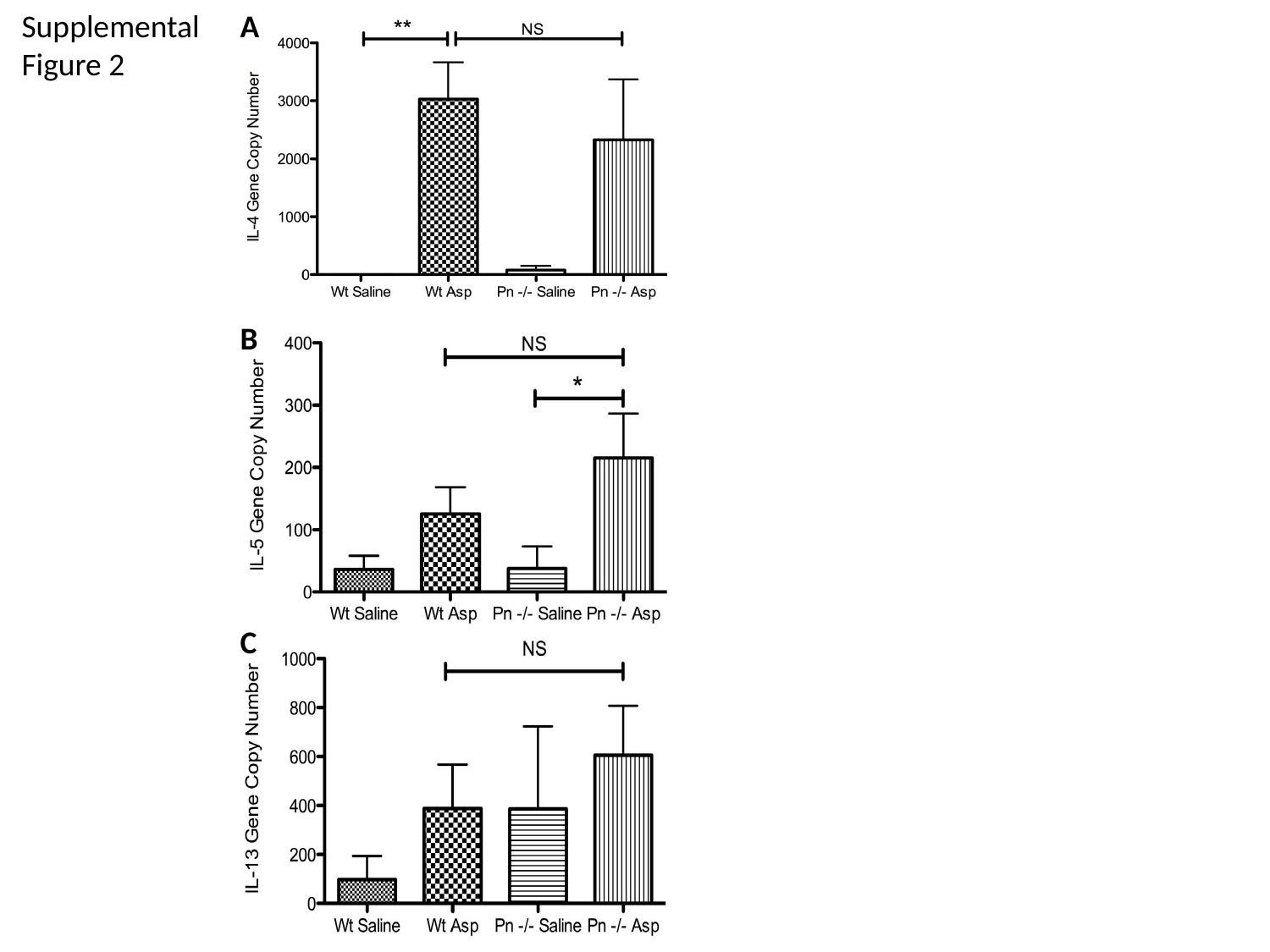

Supplemental
Figure 2
A
B
C

Supplement: Supplementary file 1 [file cea0042-0144-SD1.pptx]
